# Positive Childhood Experiences and Adult Outcomes: A Systematic Review

**DOI:** 10.1177/15248380241299434

**Published:** 2024-11-30

**Authors:** Olga Cunha, Marta Sousa, Bárbara Pereira, Marina Pinheiro, Ana Beatriz Machado, Sónia Caridade, Telma Catarina Almeida

**Affiliations:** 1 Psychology Research Center (CIPsi), University of Minho, Braga, Portugal; 2 William James Center for Research (WJC), ISPA—University Institute, Lisbon, Portugal; 3 Egas Moniz Center for Interdisciplinary Research (CiiEM), Egas Moniz School of Health & Science, Caparica, Almada, Portugal

**Keywords:** positive childhood experiences, adverse childhood experiences, benevolent childhood experiences, childhood adversity, counter-ACEs

## Abstract

Although positive childhood experiences (PCEs) may serve as protective factors against the negative consequences of childhood adversity, they have been less extensively studied. However, more recently, there has been a growing interest in understanding the role of these experiences. This systematic review aims to address this research gap by systematizing the existing literature on PCEs and examining their relationship with both positive and negative outcomes. A comprehensive search of databases such as *B-On*, *PsycINFO*, *PubMed*, *SCOPUS*, and *Scielo* identified 87 studies that met the inclusion criteria. Different studies have employed various designs and samples to investigate the relationship between PCEs and adult outcomes. The findings suggest that higher levels of PCEs are consistently associated with better mental health outcomes, such as decreased depressive symptoms, anxiety, and suicidal behaviors, as well as improved psychosocial well-being, including reduced perceived stress and increased life satisfaction. Conflicting results were found for behavioral outcomes, physical health, stressful life events, and parenting and family functioning. In addition, the interaction effect of PCEs on adverse childhood experiences (ACEs) in adulthood is inconsistent. PCEs and ACEs appear to be independent sets of experiences that often coexist, with PCEs frequently not moderating the consequences of adversity on outcomes. More research with diverse samples is needed to better understand the role of PCEs.

## Introduction

Childhood experiences, whether positive or negative, establish the framework for a person’s development across their lifespan (Masten & Cicchetti, 2016). While examining individuals’ adaptive development, the focus is often on the impact of adverse childhood experiences (ACEs) rather than the significance of positive childhood experiences (PCEs; Crandall et al., 2019). However, it is vital to recognize the role of PCEs in the presence of ACEs and their potential to moderate or alleviate the adverse effects of ACEs (Crandall et al., 2019). Children’s assets and resources, such as positive family relationships, predictable routines, positive relationships at school, and positive experiences in the community, promote competent development and help counteract the negative consequences of adversity (Crandall et al., 2019; Masten & Cicchetti, 2016). Positive relationships and experiences accumulate across multiple levels, resulting in cumulative benefits for positive adjustment and adaptation (Han et al., 2023).

When referring to PCEs, it is important to explain the process of “allostasis.” This process, which involves physiological changes that help individuals cope with stressful situations, happens due to a necessity to adapt to the constantly changing environment around us while aiming to maintain physiological and behavioral stability (Boullier & Blair, 2018). After experiencing stress, individuals typically have a recovery period and return to a healthy activation level. Even if the stress is severe, they may still be able to regulate effectively with the help of protective factors from PCEs, such as a parent or caregiver who can assist in regulating the response and building the child’s resilience (Boullier & Blair, 2018). Research proves that PCEs are promotive factors for positive functioning in adulthood (Narayan et al., 2018). These experiences, known as counter-ACEs, have a positive impact on long-term development and may lead to positive outcomes for adult mental and physical health and well-being (Wright et al., 2013). However, if the stressful situations are frequent or there are no protective factors to help in recovery, it can lead to constant dysregulation with long-term consequences on the functioning of the neurological, endocrine, and immune systems (Boullier & Blair, 2018).

Although PCEs can have beneficial effects, it is essential to acknowledge that ACEs significantly influence adversity in adulthood (Almeida et al., 2021). To fully comprehend the relationship between positive and negative childhood experiences and adult outcomes, it is important to understand how resilience factors modify the path from risk exposure to adverse outcomes. In this regard, there are three general classes of resilience models: compensatory, protective, and challenge (Fleming & Ledogar, 2008).

In a compensatory model, a resilience factor opposes a risk factor, having an independent effect on the result, not influenced by the risk factor (Andersson & Ledogar, 2008). In the protective model, resources moderate or reduce the effects of a risk factor on a negative outcome. Protective factors may neutralize or weaken the effects of risks or enhance the positive effects of other promotive factors in producing an outcome (Fleming & Ledogar, 2008). In the challenge model, the association between a risk factor and an outcome is curvilinear, meaning that exposure to both low and high levels of a risk factor leads to negative outcomes. However, moderate levels of risk are associated with less negative (or positive) outcomes. This moderate exposure to risk can help individuals learn how to overcome it (Fleming & Ledogar, 2008).

Studies have shown that PCEs are associated with a reduced prevalence of chronic pain, a positive association with later cognition, the establishment of a foundation for improved family health in adulthood, and lower prenatal depression and post-traumatic stress disorders (PTSD) symptoms (Bunting et al., 2023; Craig et al., 2022; Crandall et al., 2020; Daines et al., 2021; Narayan et al., 2018). Furthermore, PCEs can counterbalance or offset the negative consequences of ACEs, thereby reducing negative outcomes (Narayan et al., 2018), mainly when ACE scores are moderate on various aspects of adult health such as cognitive and emotional well-being, psychopathology, stress during pregnancy, suicidality, and recidivism (Bunting et al., 2023; Narayan et al., 2018). However, research indicates that PCEs exert their influence largely independently of ACEs (Bunting et al., 2023). In cases where youth have experienced a high number of PCEs, the positive association between ACEs and recidivism is no longer significant (Baglivio & Wolff, 2021). Crandall et al. (2020) proposed that PCEs may directly and independently reduce anxiety in young adults. Nevertheless, this effect may be more pronounced when the number of PCEs outweighs the number of ACEs. It has been established that PCEs play a buffering role, although their impact follows a dose-response effect, meaning their effectiveness may vary depending on the quantity (Bethell et al., 2019).

Both ACEs and PCEs play a role in shaping personality development. In a study conducted by Gunay-Oge et al. (2020b), significant associations were found between almost all symptoms of different personality disorders and ACEs, even in the presence of PCEs. In this study, it was possible to conclude that PCEs reduce the risk of personality psychopathology and that these experiences do not predict histrionic, narcissistic, and sadistic traits.

From a developmental psychopathology perspective (Cicchetti & Toth, 2009), the significance of early social experiences becomes evident. These experiences encompass various aspects, including attachments formed with caregivers, relationships established with peers, teachers, and extended relatives, as well as cultivating a positive sense of self (Malekpour, 2007). They serve as the foundation for healthy relationships and the integration of social encounters in the future (Cicchetti & Toth, 2009; Masten, 2006; Waters & Cummings, 2000). In the context of early adversities like maltreatment, exposure to violence, and family dysfunction, the risk for later depression and anxiety is increased. Thus, positive self-perceptions, parental warmth, father involvement, positive peer relationships, school connectedness, and neighborhood collective take on heightened importance, reducing the risk for later depression and anxiety (Malekpour et al., 2007). They act as a protective buffer, aiding in adaptation and resilience (Luthar et al., 2015; D. Wang et al., 2021; Wright et al., 2013). Previous research has demonstrated links between childhood abuse and subsequent experiences of sexual abuse, intimate partner violence (IPV), and other forms of victimization in adulthood (Desir & Karatekin, 2019; Riedl et al., 2019). On the other hand, a high number of ACEs has been shown to lead to an increased likelihood of reoffending, while a greater number of PCEs can lower reconviction and rearrest rates, even in the presence of ACEs (Baglivio & Wolff, 2021).

Adopting a public health approach to enhance PCEs, especially among vulnerable populations with lower counter-ACEs and higher ACEs, can potentially improve lifelong health outcomes (Crandall et al., 2019). The proactive promotion of PCEs for children may reduce the risk of smoking, drinking alcohol, antisocial behavior, poor executive function, and adult depression and promote adult relational health (Bethell et al., 2019; Boullier & Blair, 2018). Joint assessment of PCEs and ACEs may better target needs and interventions and enable a focus on building strengths to promote well-being (Bethell et al., 2019).

## Current Study

While the impact of ACEs is extensively documented, more research has yet to develop into the relationship between PCEs and different outcomes, either in conjunction with or separate from ACEs (Han et al., 2023). However, in recent years, there has been a burgeoning interest within the scientific community in understanding the link between PCEs and adult outcomes, as well as the influence of PCEs on individuals’ development, particularly in the context of ACEs and in the potential protective or buffering role of PCEs against ACEs. Nevertheless, a recent body of research suggests that the impact of PCEs on outcomes may more frequently occur independently from ACEs rather than moderating the effects of adversity (Bunting et al., 2023; Han et al., 2023). This systematic review aims to systematize the existing literature on PCEs, examining the relationship between PCEs and different positive and negative outcomes in adulthood.

## Methods

The review was conducted following the Preferred Reporting Items for Systematic Reviews and Meta-Analyses (PRISMA) guidelines (Page et al., 2021), and the protocol was pre-registered with OSF REGISTRIES (reference: 10.17605/OSF.IO/7QBRK).

### Search Strategy

Systematic searches were conducted using six databases: *B-On*, *PsycINFO*, *PubMed*, *SCOPUS*, and *Scielo*, with the following search equation: (“positive childhood experiences” OR “benevolent childhood experiences” OR “childhood protective factors”) AND (impact OR consequence OR effect OR outcome OR influence). The search, initially conducted in February 2024 and later in August 2024, had no data restrictions and was carried out by two independent researchers. Reference lists from the included studies were also checked to identify relevant studies missed by the search strategy.

### Eligibility Criteria

The inclusion criteria were as follows: (a) published empirical studies that underwent a peer review process, (b) reporting findings from adults (i.e., 18 years or older), (c) written in English, Portuguese, or Spanish, (d) utilizing longitudinal or cross-sectional designs, and (e) examining the relationship between PCEs and at least one outcome in adulthood. Exclusion criteria included studies with juvenile samples or mixed samples (i.e., adults and juveniles) without separated statistical analysis and gray literature (i.e., doctoral theses and master’s dissertations, books, book chapters, and conference papers). No restrictions were placed on publication dates.

### Literature Selection Process and Data Extraction

Studies identified through systematic searches were imported into Rayyan software (Ouzzani et al., 2016). First, duplicates were removed. Second, titles and abstracts were screened based on the inclusion criteria by two independent researchers. Third, the full texts of the included studies in the titles and abstract phase were independently reviewed by the same two researchers. Fourth, a codebook was developed to extract data from all the included papers in the full-text analysis. This included information about reference details (e.g., authors and year); study characteristics (e.g., location and setting); sample characteristics (e.g., size, age, gender, and ethnicity/race); design characteristics (e.g., design type and length of follow-up); measurement characteristics (e.g., assessment measures), and outcomes, including the effect sizes of the outcomes (if available). The extraction of the information was also coded by two independent researchers. Differences between raters during the title and abstract selection phase, as well as the full-text reading phase, were discussed with a third reviewer until consensus was reached.

### Quality Assessment

The Mixed Methods Appraisal Tool (MMAT; Hong et al., 2018) was used to measure the methodological quality of all included studies. This tool includes two screening questions (e.g., “Are there clear research questions?”; “Do the collected data allow to address the research questions?”) and five items to measure the methodological quality of studies, depending on the design of the study. Each item is classified as “yes,” “no,” or “don’t know.”

## Results

### Screening and Selection of Studies

**Figure 1.**
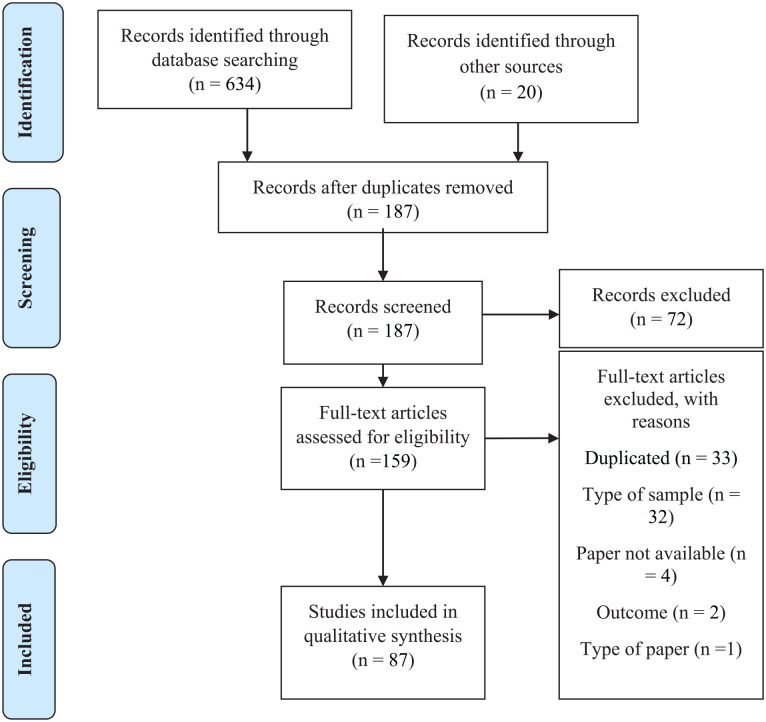
PRISMA flow diagram of the study selection process. *Note*. PRISMA = Preferred Reporting Items for Systematic Reviews and Meta-Analyses.

In total, 654 articles were gathered from the data searches, along with 18 from supplementary searches. After eliminating duplicates, 187 titles and abstracts were screened for relevance. A total of 159 studies were chosen for further examination. However, 72 additional studies did not meet the inclusion criteria. The primary reasons for exclusion were publication type, studies involving juvenile individuals, duplicated studies, and studies that did not report relevant outcomes. Ultimately, 87 studies were included in the final review. Figure 1 presents the PRISMA flow diagram, depicting the number of included studies at each stage of the selection process and the reasons for exclusion.

### Quality Assessment

Among the included articles, most were designed as quantitative non-randomized studies (*n* = 65; i.e., quantitative studies that investigate the effectiveness of an intervention or analyze other exposures without employing randomization), while the remaining were quantitative descriptive studies (*n* = 22; i.e., quantitative studies that detail the distribution of variables, with a little emphasis on exploring causal relationships or testing specific hypotheses) (see Table 1; Hong et al., 2018). Of the 87 studies, only 2 met all 5 criteria of excellence (Crandall et al., 2020; Rollins & Carndall, 2021), 11 met 4 out of 5 criteria of excellent (Almeida et al., 2021, 2022, 2023; Booth et al., 2015; Crouch et al., 2022; Hashemi et al., 2021; Hou et al., 2022; La Charite et al., 2023; Narayan, Merrick, et al., 2023; Pei et al., 2022; Woodward et al., 2023), 23 met 3 out of 5 criteria (Almeida & Costa, 2023; Almeida, Cardoso, et al., 2024; Almeida, Guarda, et al., 2024; Bethell et al., 2019; Bhargav & Swords, 2024; Brown et al., 2022; Clark et al., 2024; Crandall et al., 2021; Daines et al., 2021; Denhard et al., 2024; Feiler et al., 2023; Fentem et al., 2023; Geng, Zou, et al., 2021; Graupensperger et al., 2023; Hanson et al., 2022; Pro et al., 2020; Reese et al., 2022; Skodol et al., 2007; C. Wang et al., 2023; Zhang et al., 2021; Zheng et al., 2022; Zhu et al., 2023), 36 met 2 out of 5 criteria (Agathis et al., 2023; Ashour et al., 2024; Chung et al., 2008; Crandall et al., 2019; Crouch et al., 2023; Doom et al., 2021; Dubow et al., 2016; Geng, Li, et al., 2021; Gissandaner et al., 2024; Gunay-Oge et al., 2020b; Guo & Wang, 2023; Jadhav et al., 2023; Johnson et al., 2022; Kocatürk & Çicek, 2023; Kuhar & Kocjan, 2021; Landa-Blanco et al., 2024; Merrick et al., 2019, 2020; Miller et al., 2020; Morris et al., 2021; Narayan et al., 2021; Narayan, Frederick, et al., 2023; Nevarez-Brewster et al., 2022; Novilla et al., 2022; Orbuch et al., 2022; Rodriguez et al., 2021; Saleptsi et al., 2004; Seya et al., 2023; Shaw et al., 2023; Shevlin et al., 2022; Slopen et al., 2017; Somefun et al., 2023; Tang et al., 2023; Xu et al., 2022; Yoon et al., 2024; Yu et al., 2022), 10 met one out 5 criteria (Barnert et al., 2023; Cárdenas et al., 2022; Karatzias et al., 2020; Lee et al., 2020; Maxwell & Huprich, 2014; Mehlhausen-Hassoen & Winstok, 2019; Mešl & Rihter, 2021; Narayan et al., 2018; Rhodes et al., 2023; Starbird & Story, 2020), and 5 studies did not meet any of the 5 criteria (Chasson & Taubmen-Ben-Ari, 2023; Gunay-Oge et al., 2020a; Hakansson et al., 2018; Kosterman et al., 2011; Matos et al., 2023). The most common reasons for low quality included: participants not being representative of the target population or the sampling strategy being inappropriate for addressing the research question; measures used that were not suitable for the outcomes of interest; incomplete outcome data, with not all participants contributing to most measures; and a high risk of nonresponse bias.

**Table 1.** Main Findings.

Study	Sample	PCEs Measure	Outcomes	Findings
Agathis et al. (2023)	2,513	Self-constructed	PH	Having a strong father–child relationship was inversely associated with two risk factors among women (lifetime transactional sex (OR = 0.4) and recent age-disparate sexual relationships (OR = 0.3)). Completed or attended secondary school was inversely correlated to not knowing a partners HIV status (OR = 0.6) and having a sexual partner who is older by 10 years (OR = 0.2) in women and to infrequent condom use (OR = 0.4) in men; strong mother relationship was inversely associated with having multiple sexual partners (OR = 0.5) in women and having multiple sexual partners (OR = 0.6); have a sexually transmitted infection (STI; OR = 0.3) and being victim of sexual violence (OR = 0.3) in men. Moreover, having a caregiver monitoring and supervision was negatively correlated to having multiple sexual partners (OR = 0.7), infrequent condom use (OR = 2.4), having an STI (OR = 2.1) in women, and a STI in men (OR = 3.2).
Almeida and Costa (2023)	171	BCEs	PO	Individuals with fewer PCEs experience increased anxiety about abandonment (general population: *r* = −.355; sex offenders: *r* = −.229) and struggle to rely on others when necessary (general population: *r* = .389; sex offenders: *r* = .227).
Almeida et al. (2021)	1,886	BCEs	SDF; SLE	Participants who were married, had higher education, were employed, retired, or students, and were older, scored higher on the PCEs compared to others. The total PCE score was negatively related to the incidence of ACEs (*r* = −.471).
Almeida et al. (2022)	424	BCEs	MH	PCEs were negatively correlated with affective lability (*r* = −.263), depression/anxiety (*r* = −.228), depression/elation (*r* = −.244), and anger (*r* = −.430). PCEs did not moderate the relationship between child maltreatment and affective lability.
Almeida et al. (2023)	2,310	BCEs	MH	PCEs were negatively correlated with affective lability (*r* = −.261), depression/anxiety (*r* = −.270), depression/elation (*r* = −.232), and anger (*r* = −.217).
Almeida, Guarda, et al. (2024)	2,310	BCEs	PO	PCEs were positively correlated with perspective taking (*r* = .073) and emotional concern (*r* = .120) and negatively with personal distress (*r* = −.067) in the community sample and positively correlated with perspective taking (*r* = .156) and emotional concern (*r* = .165) in forensic sample. An interaction effect between child maltreatment (CM) and PCEs was found only for personal distress (empathy subscale) among justice-involved individuals (*b* = 0.005).
Almeida, Cardoso, et al. (2024)	1,541	BCEs	SDF; BO	No differences were found between genders on PCEs. PCEs were negatively correlated with total aggression (*r* = −.254; *r* = −.146), physical aggression (*r* = −.214; *r* = −.154), verbal aggression (*r* = −.132; *r* = −.096), anger (*r* = −.166; *r* = −.120), and hostility (*r* = −.295; *r* = −.114) in women and men. Furthermore, moderation analyses clarified the moderating effect of PCEs on the relationship between ACEs and aggression in women (*b* = 0.16) and between ACEs and anger in both sexes (men: *b* = −0.08; women: *b* = 0.07).
Ashour et al. (2024)	492	BCEs	MH; PO	PCEs showed a negative correlation with perceived stress (*r* = −.312), anxiety (*r* = −.323), depression (*r* = −.349), and sleep disturbance (*r* = −.266) and positive with resilience (*r* = .344).
Barnert et al. (2023)	1,854	Self-constructed	BO	Children of parents with more ACEs are at higher risk of arrest during adolescence and young adulthood, even after considering their parents’ PCEs.
Bethell et al. (2019)	6,188	PACEs	MH; PO	Fewer PCEs are linked to a higher prevalence of adult depression and poor MH (high PCEs: OR = 0.28; medium PCEs: OR = 0.50), while more PCEs are associated with increased social and emotional support (high PCEs: OR = 3.53; medium PCEs: OR = 1.31).
Bhargav and Swords (2024)	321	BCEs	MH; PO	PCEs had a significant negative relationship with psychological distress (*r* = −.391) and suicide ideation (*r* = −.408), and a significant positive relationship with resilience (*r* = .423). The association between ACEs and poor MH was stronger among those with fewer PCE (*r* = .230 vs. *r* = .150).
Booth et al. (2015)	185	RFQ	PO	PCEs associated with higher life satisfaction (*b* = 0.567).
Brown et al. (2022)	140	PCE	PO	Higher PCEs were associated with lower burnout (β = −.197), while they did not significantly predict compassion satisfaction.
Cárdenas et al. (2022)	208	BCEs	MH	Higher PCEs were associated with lower depressive symptoms at antepartum (*r* = −.21), newborn sessions (*r* = −.26), and 6 months postpartum (*r* = −.27). Peripartum stage did not moderate the association between PCEs and depressive symptoms.
Chasson and Taubmen-Ben-Ari (2023)	392	BCEs	P; PO	PCEs were linked to less intrusive thoughts (phase 1: *r* = −.16; phase 2: *r* = −.18) and dissociative experiences (phase 1: *r* = −.11; phase 2: *r* = −.12) and high maternal self-efficacy (phase 2: *r* = .13), role satisfaction (phase 2: *r* = .19), and self-compassion (phase 1: *r* = .28; phase 2: *r* = .25). However, the association between PCEs and maternal self-efficacy was mediated by self-compassion and dissociative experiences.
Chung et al. (2008)	1,476	Self-constructed	MH	Three (OR = 0.43) and four (OR = 0.42) PCEs and in specific positive maternal relationships (OR = 0.70) and often told “great” (OR = 0.73) were associated with lower rates of depressive symptoms.
Clark et al. (2024)	292	BCEs	MH, PO, BO	PCEs were negatively associated with anxiety problems (*r* = −.179), trait anxiety (*r* = −.186), PTSD (*r* = −.202), depression problems (*r* = −.173), depression (*r* = −.194), substance use (*r* = −.209), various personality traits/personality disorders (disinhibition (*r* = −.196), psychoticism (*r* = −.209), avoidance (*r* = −.255), detachment (*r* = −.218), and borderline personality (*r* = −.142), antisocial behavior during pregnancy (*r* = −.282). PCEs were negatively associated with the latent HiTOP dimensions: general psychopathology (β = −.268), thought problems (β = −.284), internalizing symptoms (β = −.197), detachment (β = −.249), disinhibited externalizing behavior (β = −.352), antagonistic externalizing behavior (β = −.175), and antisocial behavior during pregnancy (β = −.119). No significant associations were found between PCEs and fear, distress, or substance use.
Crandall et al. (2019)	246	BCEs	MH, PH, CF; PO	PCEs were associated with lower stress (*b* = −0.15), depression (*b* = −0.10), and sleep difficulties (*b* = −0.28), and higher scores for executive functioning (*b* = 0.08), locus of control (*b* = 0.17), forgiveness (*b* = 0.21), gratitude (*b* = 0.27), family closeness (*b* = 0.10), and daily fruit and vegetable consumption (*b* = 0.12). PCEs neutralize the effect of ACEs on health.
Crandall et al. (2020)	489	BCEs	MH, BO; PO	PCEs were associated with risky sex (β = −.14), depression (β = −.13), anxiety (β = −.12), substance abuse (β = −.14), and body image (β = .16). When accounting for ACEs, PCEs remained predictive of less risky sex (β = −.12), reduced depression (β = −.11), and less substance abuse (β = −.12), PCEs were not predictive of reduced anxiety. PCE also predicted a more positive body image (β = .15).
Crandall et al. (2021)	206	BCEs + PCE	MH, PH	More PCEs were associated with a reduced likelihood of smoking, suicidal thoughts, and emotional/cognitive health problems and with an increased probability of eating fruits and vegetables and engaging in physical activity. After accounting for ACEs, more PCEs are linked to a lower probability of having suicidal thoughts or behaviors (OR = 0.12) and emotional/cognitive health problems (OR = 0.11).
Crouch et al. (2022)	5,407	Self-constructed	SDF	People of color were less likely to be supported by friends (OR = 0.73), have an adult who took interest in them (OR = 0.45), and have a family who stood by them during difficult times (OR = 0.64). Education and poverty were significantly associated with feeling safe and protected (OR = 0.67 and 0.52), supported by friends (OR = 0.70 and 0.38), and having a family that stood by them during difficult times (OR = 0.74 and 0.49).
Crouch et al. (2023)	671	PACEs	MH	PCEs did not affect the relationship between ACEs and marijuana use during lactation.
Daines et al. (2021)	1,030	BCEs	FF	PCEs were positively associated with all four family health domains, namely family social and emotional health processes (*b* = 0.24), family healthy lifestyle (*b* = 0.26), family health resources (*b* = 0.25), and family external social supports (*b* = 0.31). PCEs were negatively correlated to ACEs (*b* = −0.30).
Denhard et al. (2024)	1,756	Self-constructed	SLE	High parental monitoring increased the likelihood of seeking services (OR = 1.79), whereas a strong father–child relationship was negatively associated with seeking services (OR = 0.45).
Doom et al. (2021)	502	BCEs	MH, PO	Higher PCEs were linked to fewer COVID-19 stressors (*r* = −.25), less loneliness (*r* = −.29), greater social support (*r* = .24), lower perceived stress (*r* = −.35), fewer symptoms of anxiety (*r* = −.30) and depression (*r* = −.35), and higher subjective socioeconomic status ( SES; *r* = −.30). After controlling for current stress, social support, and SES, higher levels of PCEs were associated with lower depressive symptoms (β = −.39); lower perceived stress (β = −.26), and less loneliness (β = −.12).
Dubow et al. (2016)	436	Self-constructed	BO	By age 8, regular church attendance by parents (OR = 0.73) and fewer negative family interactions (OR = 0.79) reduced the risk of adult violence. For youth with at least one risk factor, adolescent protective factors (OR = 10.4; but not childhood ones) lowered the likelihood of adult violence. Adolescent protective factors serve as buffer against the negative effects of risks.
Feiler et al. (2023)	1,498	BCEs	MH; SDF	People of color, LGBTQ+ individuals, and older adults had fewer PCEs (*r* = −.13), while higher parental education was associated with more PCEs. Moderate negative correlations were observed between PCEs and emotion dysregulation (*r* = −.37), PTSD (*r* = −.35), depression (*r* = −.39), and anxiety symptoms (*r* = −.33). PCEs significantly predicted a lower likelihood of positive screens for PTSD (OR = 0.65), depression (OR = 0.59), and anxiety (OR = 0.67). Small moderation effects of PCEs on the relationships of ACEs-emotion dysregulation, ACEs-depression symptoms, ACEs-anxiety symptoms, and emotion dysregulation-PTSD symptoms.
Fentem et al. (2023)	121	BCEs	MH, BO	PCEs were negatively correlated to ACEs (*r* = −.56). PCEs are not moderators in the association between PTSD symptom severity and engagement in reckless behavior.
Geng, Li, et al. (2021)	7,218	BCEs	MH	PCEs were negatively related to PTSD symptoms (β = −.19).
Geng, Zou, et al. (2021)	7,245	BCEs	MH	PCEs were negatively associated with PTSD (*r* = −.17) and depression (*r* = −.19). PCEs were independently linked to adult insomnia (β = .09). Participants with more severe insomnia reported lower levels of PCEs. PCEs were indirectly associated with insomnia through PTSD and depression, but the direct effect remained significant. After controlling for demographics, PTSD and depressive symptoms partially mediated the overall effect of PCEs on insomnia.
Gissandaner et al. (2024)	125	BCEs	P	PCEs of caregivers (*r* = −.25) showed a significant negative association with preschool externalizing problems. However, this relationship became nonsignificant when adjusting for caregiver ACEs.
Graupensperger et al. (2023)	6,495	Self-constructed	MH	PCEs scores were inversely associated with lifetime (OR = 0.66) and current cigarette use (OR = 0.61), and positively associated with alcohol use in the past 30 days (OR = 1.12). However, among individuals who did use alcohol in the past month, PCEs were inversely associated with all indices of alcohol use patterns: total drinks (OR = 0.94), drinks per occasion (OR = 0.95), heavy episodic occasions (OR = 0.91), peak drinking (OR = 0.95).
Gunay-Oge et al. (2020a)	175	BCEs	MH, PF	PCEs were negatively associated with psychopathological symptomatology (*r* = −.25) and positively linked to life satisfaction (*r* = .42).
Gunay-Oge et al. (2020b)	341	BCEs	MH	All PD symptoms except Histrionic and Narcissistic PD symptoms were negatively related with PCEs: AN: β = −.15; AV: β = −.20; BD: β = −.20; DE: β = −.16; DE: β = −.26; OC: β = −.18; PA: β = −.23; PG: β = −.25; SD: β = −.21; SZ: β = −.29; ST: β = −.22.
Guo and Wang (2023)	2,105	BCEs	PO	PCEs had a positive correlation with flourishing (*r* = .49), presence of meaning (*r* = .24), search for meaning (*r* = .15), and an inverse correlation with perceived stress (*r* = −.22). More PCEs were associated with higher flourishing (*b* = 6.82), presence of meaning (*b* = 0.91), and searching for meaning (*b* = 0.67), after adjusting for perceived stress.
Hakansson et al. (2018)	43	TAQ	MH, P, CF	PCEs were positively associated with reflective (*r* = .70) and executive functioning (*r* = .59) and negatively associated with psychopathological symptomatology (*r* = −.56). PCEs were positively associated with higher parental reflexive function (*r* = .61).
Hanson et al. (2022)	555	BCEs	MH; PO	PCEs predict lower depression and anxiety, through executive functioning, expressive suppression, cognitive reappraisal, and thriving. Students with higher PCEs seem more resilient to life stresses.
Hashemi et al. (2021)	2,887	Self-constructed	MH; SDF	People with ACEs had the highest risk of reporting poor MH or disability, regardless of whether they also experienced PCEs. Women with lower incomes and with primary or secondary education reported fewer PCEs.
Hou et al. (2022)	1,816	BCEs	MH; PO	Individuals with higher PCEs were less likely to experience psychological distress (including US, OR = 0.33, depressive symptoms, OR = 0.22, and suicidal ideation, OR = 0.32). The combined effect of ACEs and PCEs (High-Both group) could play a protective factor in US (OR = 0.56) and depressive symptoms (OR = 0.47).
Jadhav et al. (2023)	445	BCEs	PO; BO	PCEs were negatively related to both hopelessness (*r* = −.223), emotional vulnerability (*r* = −.172), and gender discrimination (*r* = −.358). Higher levels of hopelessness were observed among those with lower PCEs (*b* = −0.156). However, PCEs do not neutralize the effect of gender discrimination.
Johnson et al. (2022)	547;1,098	BCEs	FF	Exposure to adversity, regardless of PCEs, was associated with increased levels of family dysfunction.
Karatzias et al. (2020)	275	BCEs	MH	PCEs predicted directly only complex PTSD (β = −.24).
Kocatürk and Çicek (2023)	570	PCE	PO	PCEs significantly predicted self-esteem (*r* = .52) and resilience (*r* = .27).
Kosterman et al (2011)	313	PCE	PO; MH	PCEs significantly predicted better adult functioning (β = .55) and less substance use (β = −.49).
Kuhar and Kocjan (2021)	4,847	RQ	MH; PH	Poor self-rated mental (OR = 0.46) and physical health (OR = 0.60) were less common among participants with above-median PCEs. The link between PCEs and MH outcomes and physical inactivity varied with levels of ACE exposure.
La Charite et al. (2023)	7,105	Self-constructed	MH; PH	Higher scores for all the PCE domains were associated with lower odds of reporting fair or poor general health status: peer support and healthy school climate (OR = 0.82), paternal relationship (OR = 0.83), neighborhood safety (OR = 0.84). The association between PCEs and health outcomes weakened but was not statistically influenced by exposure to ACEs.
Landa-Blanco et al. (2024)	410	BCEs	MH; SDF; PO	PCEs did not vary significantly between men and women (*d* = −0.12). The number of reported PCEs correlates positively and significantly with flourishing (*r* = .43) and all the Light Triad traits. The number of PCEs had a significant total (β = .43) and direct effect (β = .28) on flourishing scores. Faith in Humanity and Humanism, not Kantianism, mediated the relationship between PCEs and flourishing. PCEs significantly explained all Light Triad traits.
Lee et al. (2020)	4,849	Self-constructed	MH; PH	Individuals with PCEs were less likely to have depression in adulthood (OR = 0.85–0.81), health-risk behaviors, and general health problems (OR = 0.79–0.86).
Matos et al. (2023)	76	BCEs	PO	Negative correlations were found between emotional neglect and PCEs (*r* = −.600). Physical (*r* = −.251) and emotional abuse (*r* = −.252), and physical neglect (*r* = −.384) are negatively correlated with PCEs. PCEs protect individuals from ACEs and promote better health and resilience in adulthood.
Maxwell and Huprich (2014)	599	PAQ	PO	Self-esteem was correlated positively with PCEs (*r* = .32–.34).
Mehlhausen-Hassoen and Winstok (2019)	618	Self-constructed	MH	The perceived childhood experience mediated the overall impact of the violence variables on positive MH. The effect of the perceived PCEs factor on the positive-mental-health factor was strong, significant, and positive (β = −.55).
Merrick et al. (2019)	50	BCEs	PO, P	PCEs predicted lower odds of psychological distress (OR = .71). PCEs did not predict parenting stress.
Merrick et al. (2020)	101	BCEs	PH, SLE	PCEs predicted risky reproductive planning (childhood PCEs: β = −.23; adolescent PCEs: β = −.24). PCEs predicted prenatal stress life events (early childhood PCEs: β = −.21; middle childhood PCEs: β = −.22; adolescent PCEs: β = .32).
Mešl and Rihter (2021)	4,939	Self-constructed	SDF	The results indicate that adverse socioeconomic status is associated with a greater number of ACEs and a lower number of PCEs.
Miller et al. (2020)	246	BCEs	MH; CF	PCEs were associated with decreased depression (*b* = −0.10), stress (*b* = −0.17), and higher executive functioning (*b* = 0.16).
Morris et al. (2021)	109	PACEs	P; SDF	PCEs were positively correlated with nurturing parenting attitudes (*r* = .29), parent income (*r* = .32), and education levels (*r* = .33).
Narayan et al. (2018)	101	BCEs	MH; SLE; PO	Higher levels of PCEs were significantly inversely associated with depressive symptoms (*r* = −.24), PTSD symptoms (*r* = −.37), perceived stress (*r* = −.26), and SLEs (*r* = −.37) after accounting for ACEs. PCEs only significantly predicted trauma-related outcomes, prenatal PTSD symptoms (β = −.24), and SLEs (β = −.23).
Narayan et al. (2020)	101	BCEs	MH	Higher levels of PCEs directly predicted more angel memories during pregnancy (β = .34), and, indirectly, more angel memories postnatally through pregnancy memories (β = .14). PCEs did not predict ghost memories, and ACEs did not predict angel memories.
Narayan, Merrick, et al. (2023)	1,746	BCEs	MH	PCEs predicted lower levels of depression (*r* = −.510) and anxiety symptoms (*r* = −.435), suicidal thoughts and behaviors (*r* = −.513).
Narayan, Frederick, et al. (2023)	548;1,198	BCEs	PO; MH	PCEs predicted all outcomes except aggression, i.e., depression (sample 1: *r* = −.34; sample 2: *r* = −.33); anxiety (sample 1: *r* = −.44; sample 2: *r* = −.45); life satisfaction (sample 1: *r* = .43; sample 2: *r* = .44); thoughts and suicidal behaviors (sample 1: *r* = −.42; sample 2: *r* = −.42).
Nevarez-Brewster et al. (2022)	164	BCEs	MH	Higher levels of PCEs predicted better sleep quality throughout pregnancy (*b* = −0.60).
Novilla et al. (2022)	206	BCEs	MH; PO	PCEs were associated with less shame (*r* = −.436), stress (*r* = −.452), and tobacco use (*r* = −.293), but were not directly associated with depression.
Orbuch et al. (2022)	248	Self-constructed	MH; PH	Individuals who reported PCEs described feeling hopeful about the future (OR = 2.77) and reported lower smoking rates as adults (OR = 0.30). PCEs were also associated with better self-reported adult health status (OR = 2.31).
Pei et al. (2022)	1,830	BCEs	PF	PCEs were negatively correlated with “no ways to suit the important changes in life,” “all things are not going well,” “feel that there is nothing to do,” and positively correlated with “having a positive self-concept.”
Pro et al. (2020)	35,614	Self-constructed	BO	PCEs were negatively associated with predicted physical IPV (White women: β = −.031; AI/AN women: β = −.029; Black men: β = .001).
Reese et al. (2022)	482 couples	BCEs	FF	Mothers’ and fathers’ PCEs were not associated with adverse family experiences but were associated with better family health (Father PCEs: *r* = .244; Mother PCEs: *r* = .179).
Rhodes et al. (2023)	19,120	Self-constructed	MH	Total PCEs score was negatively related to MH problems. At higher levels of PCEs, the relation between ACEs and MH outcomes was attenuated.
Rodriguez et al. (2021)	214	BCEs	MH	PCEs were negatively related to depression (*r* = −.30) and anxiety (*r* = −.30). The relationship between ACEs and MH symptoms was dependent on the level of PCEs reported.
Rollins and Crandall (2021)	489	Self-constructed	MH	PCEs did not predict any of the young adult MH outcomes.
Saleptsi et al. (2004)	192	TAQ	MH	The number of PCEs showed no notable variance across groups, except for safety scores, which were notably lower in patients with PD compared to the other groups.
Seya et al. (2023)	18,899	Self-constructed	MH	Females: strong mother–child relationship was protective against moderate to severe mental distress (OR = 0.7), suicidal/self-harm behaviors (OR = 0.6), and substance use (OR = 0.6). Males: strong mother–child relationship was protective against suicidal/self-harm behaviors (OR = 0.59); strong father–child relationship was protective against suicidal/self-harm behaviors (OR = 0.4) and substance use (OR = 0.6).
Shaw et al. (2023)	311	A-CYRM	PO	PCES were related to higher levels of mental toughness (*r* = .31) and well-being (*r* = .39). PCEs predicted mental toughness (β = .30).
Shevlin et al. (2023)	624	MHFS	MH; PO	PCEs were negatively correlated with depression (*r* = −.35), anxiety (*r* = −.27), stress (*r* = −.30), and loneliness (*r* = −.53).
Skodol et al. (2007)	520	CEQ-R	MH	Specific positive achievements recorded and positive relationships during childhood or adolescence were significantly related to the probability of remission of several personality disorders. AV: total achievement experiences Hazard Ratio = 1.14; total positive relationships: Hazard ratio = 1.10; ST: total achievement experiences Hazard Ratio = 0.93; total positive relationships: Hazard ratio = 1.27; BO: total achievement experiences Hazard Ratio = 0.99; total positive relationships: Hazard ratio = 1.02.
Slopen et al. (2017)	1,147	Self-constructed	PH	PCEs were associated with cardiovascular health indirectly through education (β = .13), depression (β = .06), and social support (β = .04), and no direct effect was observed (β = −.01).
Somefun et al. (2023)	313	BCEs	MH	Participants with high PCEs had lower odds of being moderately depressed compared to their counterparts with low PCEs, but this association was not statistically significant. Mild depression: Relative Risk Reduction (RRR) = 0.82; Moderate depression: RRR = 0.41; Severe depression: RRR = 0.90.
Starbird and Story (2020)	337	BCEs	MH	PCEs had a positive relationship with narcissism (*r* = .30).
Tang et al. (2023)	1,816	BCEs	MH	Higher PCEs protected the MH of the sample (depression: OR = 0.05; suicidal ideation: OR = 0.15).
C. Wang et al. (2023)	407	BCEs	MH	PCEs were negatively associated with depression (*r* = −.29). The relation was partially mediated by regulatory emotional self-efficacy.
Woodward et al. (2023)	9,468	PCEs	PO	Bereaved individuals reported significantly lower PCEs.
Xu et al. (2022)	332	BCEs	MH; PO	Cumulative PCEs were found associated with lower risks of depression (5–6: OR = 0.22; 7–8: OR = 0.18; 9–10: OR = 0.15), anxiety (5–6: OR = 0.23; 7–8: OR = 0.28; 9–10: OR = 0.14), and loneliness (5–6: OR = 1.01; 7–8: OR = 0.39; 9–10: OR = 0.18), as well as better self-rated health (5–6: OR = 0.41; 7–8: OR = 0.19; 9–10: OR = 0.07), life satisfaction (5–6: OR = 0.29; 7–8: OR = 0.07; 9–10: OR = 0.05), and meaning in life (5–6: OR = 0.45; 7–8: OR = 0.17; 9–10: OR = 0.05).
Yoon et al. (2024)	239	Self-constructed	PH	Some PCEs (i.e., childhood happiness and supportive parental care) moderated the positive associations of childhood abuse with pain and physical functioning. Participants who endorsed high levels of childhood happiness, greater exposure to childhood abuse was associated with higher Brief Pain Inventory Pain Interference scores.
Yu et al. (2022)	9,468	BCEs	PO	Cumulative PCEs had moderate correlations (*r* = .31–.39) with all flourishing indices and domains except for a small correlation (*r* = .24) with domain “financial and material stability.” PCEs household subdimension had small correlations (*r* = .19–.28) with all flourishing indices and domains. PCEs community subdimension had moderate correlations (*r* = .30–.36) with all flourishing indices and domains, except for small correlations with domain “character and virtue” (*r* = .29), and domain “financial and material stability” (*r* = .21).
Zhang et al. (2021)	1,821	BCEs	MH	PCEs were negatively correlated with depression (*r* = −.461). The US partly mediated the link between PCEs and depressive symptoms (indirect effect = −0.47). The association between US and depressive symptoms was significantly modified by family relationships (interact effect = −0.019).
Zhan et al. (2021)	6,929	BCEs	MH; BO; SLE	PCEs were negatively linked with severity of PTSD (*r* = .17) and depression (*r* = −.19), while positively correlated with prosocial behaviors (*r* = .22). PCEs were not related to lifetime trauma exposure.
Zheng et al. (2022)	379	EMWSS	MH	PCEs significantly predicted mobile phone addiction (β = −.40), and alexithymia (β = −.48).
Zhu et al. (2023)	2,587	BCEs	P	Children whose mothers reported high scores on PCEs were less likely to have psychosocial challenges (>9 PCEs: OR = 1.24; ≤9 PCEs: OR = 1.20). PCEs somewhat mitigated the negative effects of ACEs on offspring’s total difficulties and prosocial problems (<4 ACEs: OR = 0.79; ≥4 ACEs: OR = 0.89).

*Note.* ACE = adverse childhood experience; A-CYRM = adaptation of the child and youth resilience measure; AN = antisocial; AV = avoidant; BCEs = benevolent childhood experiences; BD = borderline; BO = behavioral outcome; CEQ-R = childhood experiences questionnaire-revised; CF = cognitive functioning; DE = dependent; EMWSS = early memories of warmth and safeness scale; FF = family functioning; IPV = intimate partner violence; MH = mental health; MHFS = memories of home and family scale; OC = obsessive-compulsive; P = parenting; PA = paranoid; PACEs = protective and compensatory experiences measure; PAQ = parental attachment questionnaire; PCE = positive childhood experiences; PG = passive-aggressive; PH = physical health; PO = psychosocial outcome; PTSD = post-traumatic stress disorder; RFQ = risky families questionnaire; RQ = resilience questionnaire; SD = self-defeating; SDF = sociodemographic factors; SLE = stressful life events; ST = schizotypal; SZ = schizoid; TAQ = traumatic antecedents questionnaire; US = uncertainty stress.

### Characteristics of Included Studies

#### Study Characteristics

The articles were published between 2007 (Skodol et al., 2007) and 2024 (Almeida, Cardoso, et al., 2024; Almeida, Guarda, et al., 2024; Ashour et al., 2024; Gissandaner et al., 2024; Landa-Blanco et al., 2024; Yoon et al., 2024). The most publications were in 2023 (*n* = 28), followed by 2022 (*n* = 15), 2021 (*n* = 8), 2020 (*n* = 6), 2019 (*n* = 4), and 2018 (*n* = 2). All other years had one publication each. Almost half of the studies (*n* = 42) were conducted in the United States, 14 in China, and 7 in Portugal. The remaining were from the United Kingdom (*n* = 3), Turkey (*n* = 3), Israel (*n* = 2), Slovenia (*n* = 2), New Zealand (*n* = 1), Canada (*n* = 1), Norway (*n* = 1), India (*n* = 1), Namibia (*n* = 1), Honduras (*n* = 1), and Ireland (*n* = 1). One study was conducted in Germany, Switzerland, and Romania, and five did not specify the country (Bhargav & Swords, 2024; Denhard et al., 2024; La Charite et al., 2023; Seya et al., 2023; Somefun et al., 2023). Moreover, one study was conducted in the United Kingdom and Middle Eastern countries. Table 1 summarizes the key characteristics of the included studies.

#### Sample Characteristics

The studies varied in sample size, from 10 participants (Matos et al., 2023) to 19,120 (Rhodes et al., 2023), and in mean age, from 19.80 years (Guo & Wang, 2023; Zheng et al., 2022) to 65 years (Yoon et al., 2024). Most samples included both men and women (*n* = 60), with the exception of 11 studies with only female participants and 3 with only male participants. Additionally, most studies were community-based (*n* = 76) (see Table 1).

### PCEs Measures

Most studies (*n* = 51) assessed PCEs using the Benevolent Childhood Experiences (BCEs) scale (Narayan et al., 2018). Other studies employed alternative measures, including established questionnaires like Traumatic Antecedent Questionnaire (*n* = 2; Hakansson et al., 2018; Saleptsi et al., 2004), PCEs (*n* = 4; Brown et al., 2022; Kocatürk & Çicek, 2023; Kosterman et al., 2011; Woodward et al., 2023), Protective and Compensatory Experiences measure (*n* = 3; Bethell et al., 2019; Crouch et al., 2023; Morris et al., 2021), Risky Families Questionnaire (*n* = 1; Booth et al., 2015), Resilience Questionnaire (*n* = 1; Kuhar & Kocjan, 2021), Memories of Home and Family scale (*n* = 1, Shevlin et al., 2023), Childhood Experiences Questionnaire (*n* = 1, Skodol et al., 2007), Parental Attachment Questionnaire (*n* = 1; Maxwell & Huprich, 2014), an adaptation of the Child and Youth Resilience (*n* = 1; Shaw et al., 2023), and Early Memories of Warmth and Safeness scale (*n* = 1; Zheng et al., 2022). One study combined BCEs and PCE (Crandall et al., 2021). The remaining studies used adaptations or screening questions. The most common positive experiences included in the different PCEs’ measures can be grouped into three categories: perceived relational and internal security (e.g., having at least one safe caregiver and beliefs that provided comfort), positive and predictable quality of life (e.g., regular meals and a consistent bedtime), and interpersonal support (e.g., having a caring teacher).

### Outcomes

Mental health problems were the most common outcome (*n* = 57), followed by psychosocial (*n* = 29), physical health outcomes (*n* = 10), and behavioral (*n* = 9). Other outcomes included sociodemographic factors (*n* = 7), stressful life events (*n* = 5), parenting (*n* = 3), family functioning (*n* = 3), and cognitive functioning (*n* = 3). Mental health outcomes measured included depression or depressive symptoms (*n* = 24), anxiety (*n* = 11), PTSD or complex PTSD (*n* = 8), suicidal thoughts and behaviors (*n* = 6), mobile phone addiction or alcohol/substance use (*n* = 8), personality disorders (*n* = 6), affective lability (*n* = 2), and insomnia/sleep problems (*n* = 2). Ten studies focused on unspecified mental health problems.

Psychosocial problems refer to non-clinical psychological factors (e.g., self-esteem, well-being, gratitude, and perceived stress) and external resources (e.g., social support) affecting well-being and social functioning. Psychosocial outcomes included life satisfaction (*n* = 4), perceived stress/psychological distress (*n* = 5), presence of meaning (*n* = 4), and indicators such as burnout, loneliness, hopelessness, emotional vulnerability, self-esteem, body image issues, empathy, well-being, social support, fear of abandonment, fear, shame, and resilience. Behavioral outcomes included criminal delinquency/aggressive behavior (*n* = 6), prosocial behaviors (*n* = 1), and risky sexual behaviors (*n* = 1). Physical health outcomes included physical activity (*n* = 2), fruit and vegetable consumption (*n* = 2), and other indicators such as reproductive and cardiovascular health.

#### Mental Health

Almost all studies found that higher PCEs are related to lower depressive symptoms, anxiety, and suicidal thoughts and behaviors (see Table 1). Of the 24 studies on PCEs and depressive symptoms, only 2 did not find significant associations (Novilla et al., 2022; Somefun et al., 2023). Novilla et al. (2022) found that shame mediated the relationship between childhood experiences and depression, while Somefun et al. (2023), in a sample of young adults in South Africa, found no association (Somefun et al., 2023).

Higher PCEs were also linked to lower symptoms or remission from personality disorders (e.g., Clark et al., 2024; Gunay-Oge et al., 2020b; Saleptsi et al., 2004; Skodol et al., 2007; Starbird & Story, 2020), reduced PTSD symptoms or severity (Feiler et al., 2023; Geng, Li, et al., 2021; Narayan et al., 2018; Zhan et al., 2021), lower substance use (Clark et al., 2024; Crandall et al., 2021; Graupensperger et al., 2023; Kosterman et al., 2011; Novilla et al., 2022; Seya et al., 2023; Zheng et al., 2022), lower affective lability (Almeida et al., 2022, 2023), reduced insomnia/sleep problems (e.g., Ashour et al., 2024; Crandall et al., 2019; Geng, Zou, et al., 2021; Nevarez-Brewster et al., 2022), and lower alexithymia (Zheng et al., 2022). One study found that PCEs predicted complex PTSD symptoms but not PTSD symptoms (Karatzias et al., 2020), and another study reported that PCEs did not moderate the relationship between PTSD severity and reckless behavior (Fentem et al., 2023). The effect sizes ranged from small (<0.29; Cohen, 1988) to large (>0.50; Cohen, 1988; see Table 1).

However, not all research suggests that higher PCEs lessen the impact of ACEs on mental health. For example, Hashemi et al. (2021) found that ACEs negatively affect health even with PCEs present, and Almeida et al. (2022) reported that PCEs did not moderate the relationship between child maltreatment and affective lability. Additionally, Rollins and Crandall (2021) found that PCEs did not predict any mental health outcomes, and Crouch et al. (2023) reported no effects of PCEs on the relationship between ACEs and marijuana use during lactation.

#### Psychosocial Outcomes

All studies indicated that higher PCEs were linked to lower psychological distress/perceived stress (Bhargav & Swords, 2024; Doom et al., 2021; Hou et al., 2022; Merrick et al., 2019; Narayan et al., 2018; Novilla et al., 2022; Shevlin et al., 2023), burnout (Brown et al., 2022), loneliness (Doom et al., 2021; Shevlin et al., 2023), fear (Clark et al., 2024), anxiety related to being abandoned (Almeida & Costa, 2023), body image issues (Crandall et al., 2020), and shame (Novilla et al., 2022). Higher PCEs were also associated with increased life satisfaction (Booth et al., 2015; Gunay-Oge et al., 2020a; Narayan, Frederick, et al., 2023; Xu et al., 2022), flourishing, meaning, and search for meaning (Guo & Wang, 2023; Landa-Blanco et al., 2024; Xu et al., 2022; Yu et al., 2022), forgiveness (Crandall et al., 2019), gratitude (Crandall et al., 2019), internal locus of control (Crandall et al, 2019), resilience (Ashour et al., 2024; Bhargav & Swords, 2024; Hanson et al., 2022; Kocatürk & Çicek, 2023; Matos et al., 2023), social support (Bethell et al., 2019; Crandall et al., 2019; Doom et al., 2021), self-esteem (Kocatürk & Çicek, 2023; Maxwell & Huprich, 2014), positive self-concept (Pei et al., 2022), empathy (Almeida, Guarda, et al., 2024), hopelessness (Jadhav et al., 2023), emotional vulnerability (Jadhav et al., 2023), and well-being (Shaw et al., 2023). Mixed findings were noted for compassion: Brown et al. (2022) found no link, while Chasson and Taubmen-Ben-Ari (2023) found a positive association. The effect sizes ranged from small (<0.29; Cohen, 1988) to large (>0.50; Cohen, 1988; see Table 1).

#### Behavioral Outcomes

Findings on the relationship between higher PCEs and criminal or aggressive behavior are mixed. Barnert et al. (2023) found that higher ACEs increased the risk of arrest in adolescence and young adulthood, regardless of parents’ PCEs. Dubow et al. (2016) reported that some PCEs are associated with reduced adult violence, Pro et al. (2020) found a negative correlation between PCEs and IPV, and Clark et al. (2024) linked PCEs to decreased antisocial behavior during pregnancy. Meanwhile, Crandall et al. (2020) found that PCEs were associated with risky sexual behavior. PCEs were also negatively related to gender discrimination (Jadhav et al., 2023). Almeida, Cardoso, et al. (2024) found negative correlations between PCEs and various forms of aggression, including physical and verbal aggression, anger, and hostility. They also identified an interaction effect of PCEs on the relationship between ACEs and aggression. The effect sizes ranged from small (<0.29; Cohen, 1988) to medium (0.30–0.49; Cohen, 1988; see Table 1).

#### Physical Outcomes

Higher PCEs were associated with more consumption of fruits and vegetables (Crandall et al., 2019, 2021) and fewer general health problems/pain (La Charite et al., 2023; Lee et al., 2020; Orbuch et al., 2022; Yoon et al., 2024), specifically cardiovascular health problems (Slopen et al., 2017), and risky sexual behaviors such as reproductive non-planning (Agathis et al., 2023; Merrick et al., 2020). However, results were mixed concerning the association between PCEs and physical activity (Crandall et al., 2021; Kuhar & Kocjan, 2021). The reported effect sizes were predominantly small (<0.29; Cohen, 1988; see Table 1).

#### Stressful Life Events Outcomes

Three studies have reported a significant relationship between PCEs and the incidence of stressful life events (Almeida et al., 2021; Merrick et al., 2020; Narayan et al., 2018), while one study has shown that PCEs were not related to lifetime trauma exposure (Zhan et al., 2021). However, one study found that the link between PCEs and post-violence sexual or physical service-seeking depends on the type of PCEs (Denhard et al., 2024). The reported effect sizes ranged from small (<0.29; Cohen, 1988) to medium (0.30–0.49; Cohen, 1988; see Table 1).

#### Parenting and Family Functioning Outcomes

Higher PCEs were linked to increased maternal self-efficacy (Chasson & Taubmen-Ben-Ari, 2023), maternal satisfaction (Chasson & Taubmen-Ben-Ari, 2023), family health (Daines et al., 2021; Reese et al., 2022), parental reflective function (Hakansson et al., 2018) and fewer preschool externalizing problems (Gissandaner et al., 2024), and psychosocial challenges in children (Zhu et al., 2023). PCEs were also positively correlated with nurturing parenting attitudes (Morris et al., 2021). The effect sizes ranged from small (<0.29; Cohen, 1988) to large (>0.50; Cohen, 1988; see Table 1).

However, results varied when adjusting for ACEs. Childhood adversity was associated with family dysfunction regardless of PCEs (Johnson et al., 2022), but Zhu et al. (2023) found that PCEs could mitigate ACEs’ negative effects. Moreover, PCEs did not predict parenting stress (Merrick et al., 2019), and the link between PCEs and preschool externalizing problems became nonsignificant when adjusting for ACEs (Gissandaner et al., 2024).

#### Other Outcomes

Three studies found that higher PCEs are linked to better cognitive functioning (Crandall et al., 2019; Hakansson et al., 2018; Miller et al., 2020), most with large effect sizes (>0.50; Cohen, 1988; see Table 1). Regarding sociodemographic factors, married individuals and those employed, retired, or students had higher PCEs scores (Almeida et al., 2021; Hashemi et al., 2021), and higher education was also associated with more PCEs (Feiler et al., 2023). Moreover, females, people of color, and LGBTQ+ individuals with a lower personal income had fewer PCEs (Crouch et al., 2022; Feiler et al., 2023; Hashemi et al., 2021; Mešl & Rihter, 2021). However, one study found that PCEs did not vary significantly between men and women (Landa-Blanco et al., 2024). PCEs were positively correlated with income and education levels (Morris et al., 2021). However, findings on the relationship between age and PCEs are contradictory (Almeida et al., 2021; Feiler et al., 2023). Most of the effect sizes ranged from small (<0.29; Cohen, 1988) to medium (0.30–0.49; Cohen, 1988; see Table 1).

## Discussion

In recent years, there has been growing scientific interest in the role of PCEs on individual development, particularly in relation to ACEs, and the potential protective or buffering effects of PCEs against ACEs. This systematic review aims to systematize the literature on PCEs and their relationship with both positive and negative outcomes. It significantly contributes to the field as one of the first systematic reviews to comprehensively assess the independent and interactive role of PCEs. This systematic review thus enhances our understanding of how PCEs are related to adult adjustment, including both positive and negative outcomes.

Our results revealed a high number of publications in recent years focused on the relationship between PCEs and different outcomes, especially since 2018, reflecting the growing interest of the scientific community in these experiences (e.g., Almeida et al., 2023; Crandall et al., 2019; Narayan & Mickel, 2024). This contrasts with the study of ACEs, which has been a consistent focus of interest over many years, particularly since Felitti et al.’s (1998) studies. In addition, this reflects an imbalance in the study of both positive and negative childhood experiences and, consequently, in our understanding of the consequences these experiences have on an individual’s developmental pathway. Emphasizing positive experiences rather than solely negative ones shifts the understanding of the significant relevance of PCEs as critical factors for resilient and positive development. The proliferation of research suggests a growing awareness of the need for a balanced approach that considers both the positive and negative aspects of children’s experiences.

Nonetheless, the study of PCEs remains heterogeneous and inconsistent across countries, settings, and outcomes studied. Indeed, most studies included in this systematic review were conducted in the United States, with only a few conducted in Europe (except Portugal), Asia (except China), Africa, Oceania, and South America. As ACEs and PCEs may differ across various demographic and ethnically/culturally diverse populations (e.g., Merrick & Narayan, 2020), as well as across social contexts (e.g., Q. Wang, 2023), more studies are needed to understand the cultural nuances of PCEs outcomes. Moreover, this systematic review revealed that most studies on PCEs have drawn on community samples, with a notable paucity of clinical and forensic samples. Indeed, research has shown that forensic (e.g., Astridge et al., 2023; Hilton et al., 2019) and clinical populations (e.g., Madigan et al., 2023) have more ACEs than the general population, so it would be pertinent to recognize the existence of PCEs in these contexts and their relationship and potential interaction with ACEs. In addition, despite the considerable number of outcomes studied in the included studies, mental health is the most studied, followed by psychosocial outcomes, mainly on adverse outcomes rather than positive ones. This finding is interesting since, despite the interest in the study of PCEs, the focus is still on the adverse outcomes and the potential impact (protective or promotive) of these experiences on negative outcomes. Besides, behavioral outcomes, such as delinquent and criminal behavior, remain somewhat studied, with only five studies addressing the role of PCEs in such trajectories. Considering that ACEs have been consistently found as risk factors for delinquency and behavioral problems (e.g., Braga et al., 2028; Jackson et al., 2023) and that PCEs may act as a protective factor against health-risk behaviors (e.g., Almeida et al., 2023; Bethell et al., 2019; Narayan et al., 2018), counteracting the effects of childhood negative life outcomes (e.g., Han et al., 2023; Merrick & Narayan, 2020; Narayan et al., 2023), the study of the link between PCEs and the involvement in deviant pathways is of particular relevance, especially in terms of delinquency and crime prevention.

This systematic review found that, overall and consistently, higher PCEs were associated with fewer mental health problems (e.g., depressive and anxiety symptoms, PTSD symptoms, personality disorders, substance use, affective lability, insomnia/sleep problems, and alexithymia). These results are consistent with those found in a previous systematic review by Han et al. (2023), and are not surprising as investing in PCEs through supportive relationships, enriching environments, and opportunities for growth sets the stage for individuals to lead happier, healthier, and more successful lives as adults (e.g., Bhargav & Swords, 2024; Doom et al., 2021; Hou et al., 2022; Merrick et al., 2019; Narayan et al., 2018; Novilla et al., 2022; Shevlin et al., 2023). Results regarding other outcomes, such as psychosocial problems, although following the same pattern suggest the positive effect of PCEs, need further clarification considering the different number of outcomes included (e.g., stress/psychological distress, burnout, loneliness, fear anxiety related to being abandoned, body image issues, shame, self-esteem, resilience, and social support) and the small number of studies that study each outcome. Inconsistent findings were, however, found regarding the association between PCEs and parenting and family functioning problems (e.g., maternal self-efficacy, maternal satisfaction, family health, parental reflective function, and fewer preschool externalizing problems), behavioral problems (e.g., delinquent behavior), and stress life events. The small number of studies that studied these outcomes might explain these inconclusive findings. Therefore, future investigations should take this into account. Finally, although ACEs and PCEs may differ across various demographic variables (e.g., Merrick & Narayan, 2020), only a small number of research has been focused on such variables (e.g., Almeida et al., 2021; Feiler et al., 2023; Hashemi et al., 2021; Mešl & Rihter, 2021; Morris et al., 2021), highlighting the importance of considering such variables when analyzing the effects of PCEs.

Results from this systematic review indicate that PCEs did not always buffer the effects of ACEs (e.g., Almeida et al., 2022; Hashemi et al., 2021), consistent with previous research in the field (e.g., Han et al., 2023). For instance, Hashemi et al. (2021) demonstrated that experiencing ACEs negatively affects health regardless of the presence of PCEs. Conversely, Almeida et al. (2022) found that PCEs did not alter the relationship between childhood maltreatment and affective lability in both men and women. As noted by Han et al. (2023), these findings suggest that PCEs and ACEs are distinct sets of experiences rather than opposites, as the presence of ACEs does not prevent the occurrence of PCEs. The presence of PCEs does necessarily confer a protective or buffering effect against dysfunction, although ACEs and PCEs are often related (e.g., Almeida et al., 2021; Gunay-Oge et al., 2020a; Narayan et al., 2018). Indeed, experiencing higher levels of ACEs may be associated with lower levels of PCEs (e.g., Almeida et al., 2021; Gunay-Oge et al., 2020a), particularly when the source of ACEs and PCEs overlap (Han et al., 2023), and some individuals may experience both high levels of PCEs and ACEs (e.g., Almeida et al., 2021; Hou et al., 2022; Narayan, Merrick, et al., 2023). Therefore, considering this interrelationship between PCEs and ACEs, childhood experiences should be considered to understand individuals’ development better (see Table 2).

**Table 2.** Key Findings of the Systematic Review.

Key Findings
Most studies were conducted in the United States, with few from Europe (except Portugal), Asia (except China), Africa, Oceania, and South America.
Most studies on PCEs have drawn on community samples.
Higher PCEs were associated with fewer mental health problems and psychosocial problems.
Inconsistent findings were found regarding the association between PCEs and parenting and family functioning problems, behavioral problems, and stressful life events.
PCEs did not always buffer the effects of ACEs.

*Note*. ACE = adverse childhood experience; PCE = positive childhood experience.

### Limitations

This systematic review had some limitations that researchers should consider when designing future studies. First, data collection was limited to peer-reviewed English, Spanish, or Portuguese studies, potentially excluding valuable insights from other languages. Second, most studies were conducted in the United States and used community samples, which may limit the generalizability of the outcomes to other regions, such as Africa, South America, and Asia, as well as to clinical and forensic samples. Thus, future studies should include more diverse samples, either in terms of cultural and contextual backgrounds or in terms of settings (i.e., clinical and forensic), to better understand the specificities of PCEs and their protective or promotive role. Third, the limited number of studies examining behavioral, physical health, and sociodemographic outcomes, along with inconsistent findings regarding the link between PCEs and these outcomes, prevents us from drawing firm conclusions. Future studies should consider the role of PCEs in these outcomes, especially delinquency and criminality, and how these experiences could prevent (or not) the involvement in delinquent trajectories. Fourth, most outcomes were assessed through self-report, highlighting the importance of multimethod approaches to better capture the complexity of PCEs and their effects. Lastly, most of the included studies utilized a cross-sectional design, with PCEs being assessed retrospectively, and were of low quality, hindering a comprehensive understanding of PCEs’ role. Therefore, it is essential to conduct longitudinal studies to gain better insights into PCEs’ protective or promotive effects of PCEs. Additionally, longitudinal research is crucial for understanding the differential impact of PCEs across various developmental stages (i.e., childhood and adolescence).

### Conclusions and Implications

This review underscores the need to balance the focus between PCEs and ACEs to better understand their combined impact on development into adulthood (Almeida et al., 2023; Narayan et al., 2018). Evaluating PCEs provides crucial insights that can significantly influence mental health and social services. Mental health professionals should integrate PCEs assessments into their practice, as traditional focus has been on ACEs (Felitti et al., 1998), which are linked to adverse outcomes (e.g., Almeida et al., 2023). Incorporating PCEs into treatment plans can bolster resilience and promote positive outcomes (Narayan et al., 2018). Clinical professionals should stay informed about the most recent research on PCEs, and their constant education and training on the link between ACEs and PCEs can improve their professional skills and knowledge in identifying and intervening in risk and protective factors. Assessing PCEs offers a comprehensive view of a client’s developmental history, helping to identify protective factors. Leveraging PCEs in treatment plans can build resilience and reduce psychological distress (Hou et al., 2022). Emphasizing PCEs in individual’s lives can foster safety and trust (Narayan et al., 2018). Preventive interventions, such as programs that teach positive parenting, emotional regulation, and communication, can enhance well-being.

Educators and schoolteachers should recognize the importance of PCEs and ACEs, as PCEs are associated with fewer preschool externalizing problems (Gissandaner et al., 2024). Schools can support children by creating positive relationships and stable routines, which act as protective factors against ACEs. Programs that build positive peer connections and empathy can mitigate the adverse effects of ACEs, contributing to the individual’s healthy development. In foster care and residential centers, focusing on preventing ACEs and promoting PCEs can improve children’s overall development and well-being (Hou et al., 2022; Merrick et al., 2019). This review highlights the importance of PCEs in social services for at-risk children and those living outside their families. Supporting children in foster care, residential centers, and similar settings is vital for their development. Consistent and secure care from caregivers and staff is crucial for nurturing PCEs in youth who have faced adversity or trauma.

Additionally, culturally adapted research and interventions are essential, as PCEs and ACEs can vary significantly across cultures (Merrick & Narayan, 2020) and social contexts (Q. Wang, 2023). Professionals should tailor prevention and intervention programs to fit specific cultural and community contexts to enhance effectiveness. Further research on PCEs in diverse populations, including forensic and clinical samples, is crucial for developing effective preventive and intervention strategies (see Table 3).

**Table 3.** Implications for Research, Practice, and Policy.

Implications
Mental health professionals should integrate the assessment of PCEs into their diagnostic and therapeutic processes.
Preventive interventions may focus on promoting PCEs through programs that teach positive parenting techniques, emotional regulation, and effective communication, significantly contributing to a child’s well-being.
Educators and teachers should be educated to recognize the importance of both PCEs and ACEs in educational settings.
Creating and maintaining supportive environments for children in foster care, residential centers, and other protective settings is crucial for fostering individual development.
It is important to conduct more research on PCEs in diverse populations, including forensic and clinical samples.

*Note*. ACE = adverse childhood experience; PCE = positive childhood experience.

In conclusion, the findings from our review suggest that studying PCEs is necessary for promoting healthier developmental trajectories in individuals’ lives. Integrating the raising of PCEs into clinical, educational, or social practice can better support individuals in achieving more positive outcomes despite adversities and foster long-term resilience and well-being.
